# An Exploration Into the Measurement and Reporting of Food Firmness and Hardness

**DOI:** 10.1111/jtxs.12873

**Published:** 2024-11-18

**Authors:** Andrew J. Rosenthal

**Affiliations:** ^1^ University of Nottingham Sutton Bonington UK

**Keywords:** firmness, force, hardness, measurement, reporting, stress

## Abstract

Hardness is a commonly reported food property, measured by compression at a high strain where the food structure breaks. Hardness should not be reported for foods that deform without breaking. Firmness is an intermediate level of hardness associated with nondestructive compression, at strains typically around 0.1. Sensory perception enables accurate classifications of hardness. Conversely (and perhaps counterintuitively), instrumental measurements of hardness while often precise are not necessarily accurate. The outcome depending on the test protocol, whereby the geometry of the test apparatus, the speed of the test and the degree of deformation all influence the result. Ambiguity occurs in how instrumental measurements of hardness are reported, with some authors using stress, while others use force.

## Graphical abstract

Uncertainty occurs with the use of the terms hardness and firmness in food texture literature. Ambiguity also exists in how instrumental measurements of hardness and Firmness are reported, with some authors using stress—while others use force.

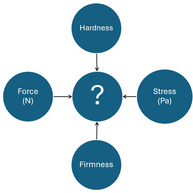

## 1 Introduction to the Hard‐Soft Dimension

Defining food texture terms is essential for understanding and communication, especially as words can take on different meanings in related disciplines, this is well illustrated by Peleg (1983) who considers a variety of scientific and linguistic uses of the word “hardness.” By way of definition the hard‐soft dimension of food texture relates to the stress or force required to break a food (Rosenthal and Chen 2023). Hardness is widely reported in the food texture literature, though the way it is recorded varies, with many researchers providing quantitative values in units of force (N), whereas other authors use stress (Pa). Of course stress is takes the contact area into account, for as Muller (1973) observes “if I sit on a chair—all is well, if I sit on a pin—all is not well”; in both cases my weight is the force, but the area over which that force is exerted makes a difference to the experience. However, there are situations in which the area of contact is difficult to determine, such as when biting or squeezing between fingers.

The widely used testing protocol texture profile analysis (TPA) has a value called “hardness” (note “quotation marks” as TPA terms are misleading). TPA is only mentioned because of its extensive use in the food texture literature, yet the intention of this short communication is to consider the advantages and disadvantages of how hardness is measured and reported. In the original TPA protocol, “hardness” was reported as a force, obtained with a die of defined geometry, thus the conversion to stress was simple (Friedman, Whitney, and Szczesniak 1963). However, the TPA protocol has been extensively modified (Nishinari, Fang, and Rosenthal 2019; Peleg 2019) with changes to strain, strain rate and contact geometry. The original TPA protocol applied a 0.75 strain and the maximum force, or the first significant break was taken as the “hardness.” Later Pons and Fiszman (1996) suggested that any break before the end of the compression should be called fracturability while hardness was the peak height at the end of the first compression.

**FIGURE 1:**
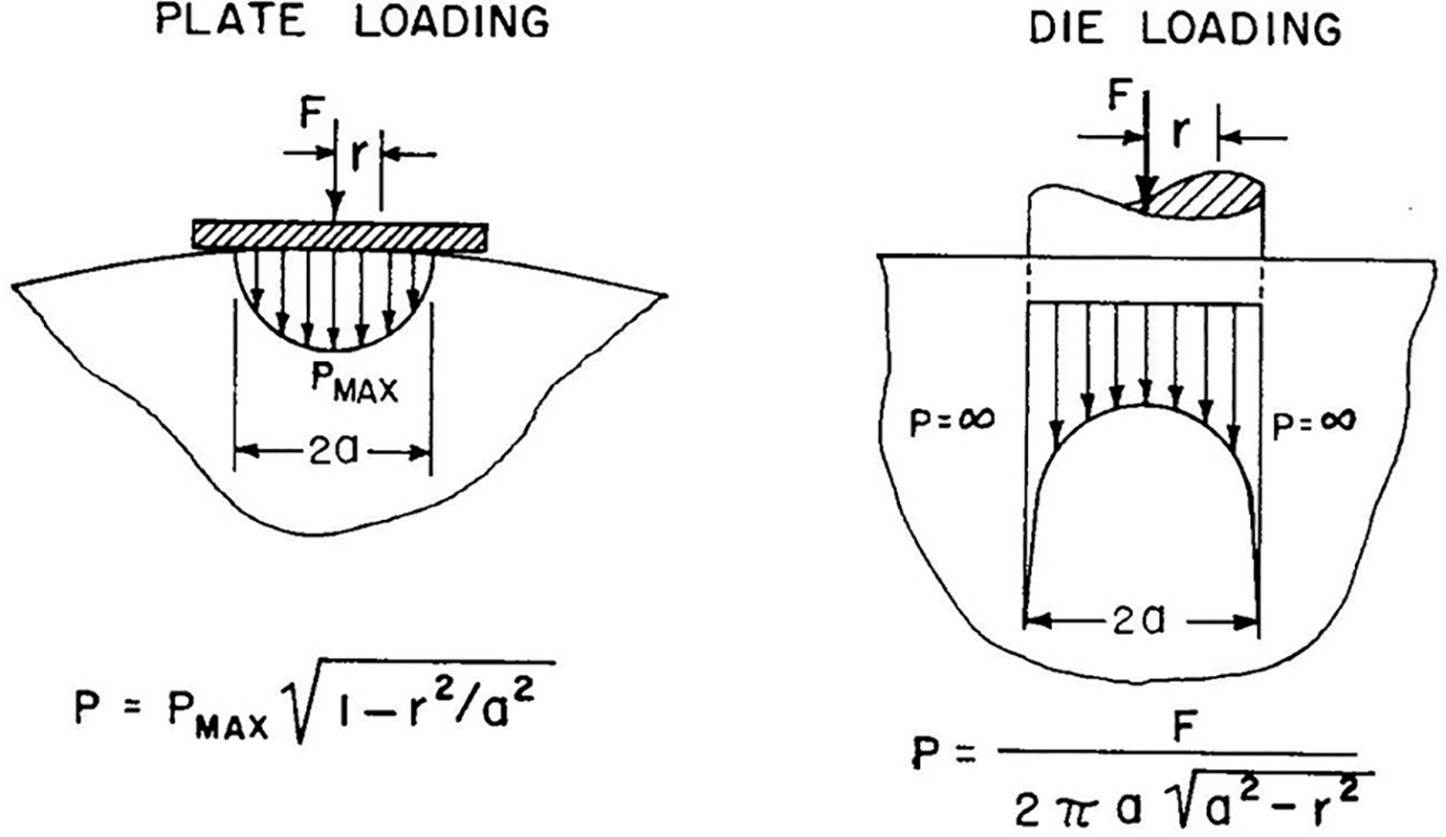
Differences between plate and die loading, from Mohsenin (1970).

Differences between the force distribution in plate and die loading are illustrated in Figure 1, from which we see that neither exerts a constant stress distribution across contact area. With plate, loading the area of contact is ill defined and increases during compression.

Correct operation of a texture analyzer is of course essential in obtaining valid data and the reader is directed to Plummer (2024) for an excellent expose on the workings of such devices.

## 2 Discussion

### 2.1 Contact Geometry

Except for gels, which can be cast into molds, few solid foods have parallel plane surfaces. Thus, only small dies can achieve complete contact at the start of compression.

As already implied only die loading has a defined contact area. Yet Figure 1 shows that the stress at the perimeter is infinite, resulting in shear. If hardness is a measure of compressive forces, then using a die is flawed. A rarely used, yet clever solution separates shear and compressive forces through repeated tests with dies of different diameters (Bourne 1966; Peleg and Gòmez Brito 1975). While plate loading does not have a shear component, the initial contact area is ill defined; moreover it increases in an unpredictable way as the sample is compressed. Thus, with plate loading we cannot represent the results as stresses and must resort to force.

Bizarrely, during a compression test we generally focus on the food surface nearest the measuring device (load cell) and totally ignore how the sample is supported. We assume that the internal stresses in the food arise entirely from the test geometry. Using very low forces, Rosenthal (2016) compared die loading of gelatin spheres on a flat base plate and supported in a mold similar to that in which it was cast. Differences in modulus resulted from the two supporting regimes. If differences arise from small forces, how much greater might we expect differences in hardness, where the material is deformed to its breaking point?

So far, we have talked about instrumental measurements of hardness, but we should remember that texture is fundamentally a sensory phenomenon, and we perceive hardness through manual handling as well as between our teeth. Haptic sensations take no account of contact area, when we squeeze fruit to gage ripeness, we feel a force, the contact area being irrelevant as we interpret the magnitude subconsciously. Such gentle squeezing is associated with the term “firmness” which the International Standard 5492 on Sensory Analysis Vocabulary (ISO 2009) describes as a moderate level of hardness. In terms of gentle squeezing our tactile acuity has been measured with fine, hair like springs, which provide a perceivable force as opposed to a stress (Aktar et al. 2015). From an instrumental point of view, such gentle squeezing is of a magnitude akin to that used to measuring a modulus, rather than the destructive compression associated with biting and hardness. Researchers have measured bite force by inserting strain gages between the teeth (Takahashi and Nakazawa 1987; Mioche, Peyron, and Auroy 1993; Nakazawa and Togashi 2000). We perceive hardness as a force, taking no account of the irregular surface geometry of our teeth.

### 2.2 Speed of Test

Stressed materials generally relax. A criterion for undertaking modulus tests is that we compress at a speed that is slow enough to allow relaxation to occur. However, if we compress a material at a speed greater than its ability to dissipate the energy, then the stresses build up. The extreme situation is an impact test where the force is applied so rapidly that the material cannot relax. In TPA (with 0.75 strain) there is a logarithmic increase in “hardness” with speed up to 2 mm s^−1^ (Rosenthal 2010). Just as with modulus testing, hardness testing might be better served if the test speed allowed complete relaxation, though the long test duration might frustrate many researchers.

### 2.3 Degree of Deformation

If a small load is applied to a material, stresses develop in the internal structure. If that load is removed and the material is elastic it will return to its original dimensions. Squeezing fruit to gage firmness or measurement of modulus are in this category of test. In contrast, large deformation tests are generally destructive. Prior to breakage there may be less obvious irrecoverable structural changes leading to partial recovery when the load is removed.

#### 2.3.1 Small Deformation (To Measure Firmness)

Instrumental tests on fruit firmness use low levels of deformation, with strains typically around 0.1 (Rivera et al. 2021) and the slope of the force‐distance curve (“hardness slope”) correlates well with sensory measurements of fruit firmness (Rivera et al. 2023). When we squeeze fruit to gage ripeness, it would be counterproductive to cause damage, thus strains of 0.1 generally allow elastic recovery. Generally small deformation tests to measure firmness are undertaken at slow speeds.

#### 2.3.2 Large Deformation (To Measure Hardness)

In characterizing hardness, we are interested in the point at which the material collapses, fractures, breaks. Catastrophic breakage generally develops through stress concentration at a structural imperfection. Once breakage commences, crack propagation continues across the sample resulting in structural break down (van Vliet and Primo‐Martin 2011). Materials which undergo this kind of behavior generally store some of the applied energy when stressed, sometimes deforming, but then fracturing when a limit is reached. As the stored energy focuses at a weak point in the structure, maybe it is wrong to associate the force with the contact area of the test geometry, in which case reporting a breaking stress is inappropriate. It should be noted that not all foods undergo rupture when compressed, rather they may deform, squash or undergo squeeze flow, and thus it would be wrong to assign the maximum force/stress as hardness for such foods?

Perhaps to avoid hitting the base‐plate in TPA, Friedman, Whitney, and Szczesniak (1963) had the die cycle to within 1/8‐inch of the base‐plate. With a standardized 1/2‐inch sample this equated to a strain of 0.75. Thus, a pragmatic solution becomes the definition. Pragmatism may also be the basis by which other researchers change the strain they use (if their instruments cannot cope with 0.75 strain, they may just reduce the deformation). Several researchers have shown that TPA “hardness” appears to increase with strain (Bourne and Comstock 1981; Rosenthal 2010), though redefining the strain used in the test does not change any inherent material property.

### 2.4 Purpose of the Measurement

Individuals can consistently order foods in magnitude of hardness. Counterintuitively, instrumental measurements of hardness while often precise and reproducible, maybe less accurate as they are method dependent. One single method is unlikely to enable hardness measurements for the entire range of foods available. Of course, if one protocol is followed for comparative purposes (e.g., quality assurance, product development, or selection of fruit/vegetable genetic variants, etc.) instrumental results are precise and discerning of differences. In its original form, TPA was correlated with sensory measurements, though to reproduce the test conditions with an obsolete instrument is difficult. Moreover variation in raw material and changes in biological materials on storage make the idea of reference values for food hardness questionable (Morrell and Rosenthal 2019).

## 3 Conclusions

Instrumental measurements and reporting hardness are not straightforward and may not be appropriate for all solid foods.

Neither die nor plate loading have isotropic force distributions. Yet, two die measurements can resolve this (Peleg and Gòmez Brito 1975), allowing shear free, *stress* values to be reported.

Rather than focusing on a point value, the shape of the stress–strain curve, enables a more meaningful gage of *firmness* (as a *stress*) at a strain of 0.1 (when compressed slowly—particularly if two die measurements are used). The shape of the stress–strain curve also provides measurement of a breaking *force* at a higher strain, as well as discounting the idea of *hardness* for foods that deform or flow rather than break.

## Author Contributions

**Andrew J. Rosenthal:** conceptualization, writing – review and editing.

## Ethics Statement

The author has nothing to report.

## Conflicts of Interest

The author declares no conflicts of interest.

## Data Availability

Data sharing not applicable to this article as no datasets were generated or analysed during the current study.
